# Supplementary material for: Do pay-for-performance schemes improve quality in community pharmacy? A mixed-methods study exploring stakeholder perspectives on implementation of the nationwide Pharmacy Quality Scheme (PQS) in England?
Source: PLoS One. 2025 Apr 16;20(4):e0319215. doi: 10.1371/journal.pone.0319215 (PMC12002447; doi:10.1371/journal.pone.0319215)
Supplement: Attachment — Submitted filename: PQS response to reviewers 121124.docx [file pone.0319215.s002.docx]

**PLOS ONE PQS paper**

**PONE-D-24-37890 – response to reviewer comments**

**Do pay-for-performance schemes improve quality in community pharmacy? A mixed-methods study exploring stakeholder perspectives on implementation of the nationwide Pharmacy Quality Scheme (PQS) in England?**

| **Comments** | **Author response** |
| --- | --- |
| **Journal comments:** |  |
| When submitting your revision, we need you to address these additional requirements.  1. Please ensure that your manuscript meets PLOS ONE's style requirements, including those for file naming. The PLOS ONE style templates can be found at  [https://journals.plos.org/plosone/s/file?id=wjVg/PLOSOne_formatting_sample_main_body.pdf [journals.plos.org]](https://urldefense.com/v3/__https:/journals.plos.org/plosone/s/file?id=wjVg*PLOSOne_formatting_sample_main_body.pdf__;Lw!!PDiH4ENfjr2_Jw!FC3X_K0x8iHRf1M7tFfuPQkx11hDsSBn2SQBNyDZg8WzBqTv4mqwX5bEjXYCxkTjMZ_1gixs6MTWtXekRv22elhmCHM8Gw$) and  [https://journals.plos.org/plosone/s/file?id=ba62/PLOSOne_formatting_sample_title_authors_affiliations.pdf [journals.plos.org]](https://urldefense.com/v3/__https:/journals.plos.org/plosone/s/file?id=ba62*PLOSOne_formatting_sample_title_authors_affiliations.pdf__;Lw!!PDiH4ENfjr2_Jw!FC3X_K0x8iHRf1M7tFfuPQkx11hDsSBn2SQBNyDZg8WzBqTv4mqwX5bEjXYCxkTjMZ_1gixs6MTWtXekRv22elhd7jukaA$) | This has been addressed – but not using track changes. ✓ |
| 2. Please provide additional details regarding participant consent. In the ethics statement in the Methods and online submission information, please ensure that you have specified (1) whether consent was informed and (2) what type you obtained (for instance, written or verbal, and if verbal, how it was documented and witnessed). | Lines 144-149 changed to: “University ethics committee approval was granted. Participants were provided with a participant information sheet PIS and gave informed consent, which was recorded either in the form of a signed consent form, returned via email, or through verbal consent, recorded in an audio file stored separately from interview data (most opted for this option).” |
| 3. Thank you for stating the following financial disclosure:  “NHS England funded the 'Independent evaluation of the quality payments scheme (QPS) and pharmacy quality scheme (PQS) 2017-2021' in September 2022.  Total funding: £177,540 (£75,214 to the University of Manchester, the rest to ICF)”  Please state what role the funders took in the study.  If the funders had no role, please state: "The funders had no role in study design, data collection and analysis, decision to publish, or preparation of the manuscript."  If this statement is not correct you must amend it as needed.  Please include this amended Role of Funder statement in your cover letter; we will change the online submission form on your behalf. | We have added this to the cover letter: “The study team were independent of the funders, but we did meet regularly to discuss study design, data collection and analysis, to ensure accuracy. The funders did not influence our analysis, interpretation, decision to publish, or preparation of the manuscript; they did however have sight of the manuscript prior to submission, again with the opportunity to comment on accuracy.” ✓ |
| 4. In the online submission form, you indicated that your data is available only on request from a third party. Please note that your Data Availability Statement is currently missing the name of the third party contact or institution / contact details for the third party, such as an email address or a link to where data requests can be made. Please update your statement with the missing information. | We have removed the word “Data are available from the University's Institutional Data Access / Ethics Committee (contact via ICF) for researchers who meet the criteria for access to confidential data.” From the Data Availability – where we ticked “No – some restrictions apply”. This is because we did not gain consent for any data sharing or third party access. ✓ |
| 5. Please include a separate caption for each figure in your manuscript. | There is only one figure in this manuscript, inserted as a separate file; the place for insertion is clearly identified. ✓ |
| 6. Please review your reference list to ensure that it is complete and correct. If you have cited papers that have been retracted, please include the rationale for doing so in the manuscript text, or remove these references and replace them with relevant current references. Any changes to the reference list should be mentioned in the rebuttal letter that accompanies your revised manuscript. If you need to cite a retracted article, indicate the article’s retracted status in the References list and also include a citation and full reference for the retraction notice. | We have checked the list of references. The only change we have made is to correct the author of reference 9: Department of Health. (line 616) ✓ |
|  |  |
| **Reviewer comments** |  |
| Reviewer #1: Thank you for the opportunity to review this manuscript. This is a very well written manuscript and worthy of publication. | Thank you! |
| • Line 120 What do the authors mean by advanced services? | There are essential services which all community pharmacies provide, and then there are advanced services which community pharmacies can choose to provide. PQS fell under these. We have added a footnote 2 to explain this:  “Under the Community Pharmacy Contractual Framework, advanced services are those which community pharmacies can choose to offer; as opposed to essential service which are offered by all.” (line 120) ✓ |
| • Line 124 For the international reader, please explain what is meant by ‘distance selling pharmacies’. | The General Pharmaceutical Council (GPhC), the pharmacy regulator in Great Britain who regulate/ register pharmacy professionals and pharmacy premises defines Distance Selling Pharmacy as “a type of pharmacy that works exclusively at a distance from patients. DSPs include mail order and internet pharmacies that remotely manage patients’ medicine logistics and distribution.”  In DSPs there can be no face-to-face contact between patients and the pharmacy staff for the delivery of essential services, but patients could access the pharmacy for non-essential services, such as advanced services. We have added “(often online/ mail order pharmacies which cannot offer essential services face-to-face)” in lines 124-125. ✓ |
| • Line 144 The information of the duration of interviews should be in the results section. | 25-60 minutes has been moved to the results, line Added in brackets in line 225 ✓ |
| • Line 146 What is meant by GDPR?5 | Added in full in line 147: “General Data Protection Regulation” ✓ |
| • Lines 168 The overview of PQS should be moved to the introduction as it does not appear to be a result of the current study | We considered the placing of this carefully prior to submission. Whilst the first two paragraphs could be incorporated into the introduction, the remainder of this section does report insights from our quantitative analysis. We believe that whilst overarching principles for the PQS are placed in the introduction, we believe that the detail which then relates to the quantitative analysis is better placed here. ✓ |
| • What is meant by ‘banding’. This may not be clear for the international reader. | The meaning of banding is explained in lines 212-214: “… pharmacy banding (introduced in 2021/22), the latter being based on the pharmacy’s number of items dispensed annually. Banding thresholds are displayed in Table 3, …” We have made no change. ✓ |
| • Table 4 is confusing | We have made changes to clarify Table 4. These include enhancing the headings to clearly indicate that the themes reflect stakeholders’ views on the PQS and how these align with variables in the Harrington framework. To avoid repetition, we focused on specific variables in the Harrington framework, omitting domain names. Lastly, we added a note below the table stating: “The themes identified in this PQS evaluation were mapped to the relevant variables in the Harrington et al. framework". ✓ |
| • Line 313 This quote was already stated in Line 304 | Apologies, we have deleted the second quote in lines 322-326. ✓ |
| • Line 546 Suggest not to use ‘etc’. | Removed in what is line 555 in the revised manuscript. ✓ |
| • Congratulations on a well-written manuscript. | Thank you, we really appreciate your helpful comments and your positive feedback. ✓ |
